# Influence of Health System Affiliation and Pain Manifestation on Advanced Oral Cavity Squamous Cell Carcinoma Risk: A Retrospective Cohort Study in a Latin American Population

**DOI:** 10.3390/dj12120383

**Published:** 2024-11-26

**Authors:** Alejandro I. Díaz-Laclaustra, Efraín Álvarez-Martínez, Carlos M. Ardila

**Affiliations:** 1 Department of Basic Sciences, Faculty of Dentistry, Universidad de Antioquia, Medellín 050010, Colombia; isbet.diaz@udea.edu.co; 2 Maxillofacial Surgery Program, Faculty of Dentistry, Universidad de Antioquia, Medellín 050010, Colombia; efrain.alvarez@udea.edu.co; 3 Biomedical Stomatology Research Group, Faculty of Dentistry, Universidad de Antioquia, Medellín 050010, Colombia

**Keywords:** oral cavity squamous cell carcinoma, health system plan, pain management, cohort study

## Abstract

**Background/Objectives:** Oral cavity cancer, a subtype of head and neck cancer, is one of the most common malignancies globally. This study assessed the influence of health system affiliation and pain manifestation on the risk of advanced oral cavity squamous cell carcinoma (OCSCC) in a Latin American population. **Methods:** In this retrospective cohort study, we analyzed medical records from 2015 to 2016, including data from the past 19 years, of 233 patients with OCSCC treated at a public hospital in Medellín, Colombia. Sociodemographic and clinical variables were evaluated, and multivariate regression models incorporated variables significant in bivariate analysis. **Results:** Among 233 patients, 196 (84.1%) had advanced OCSCC. The sample had a mean age of 63 ± 13 years, 53.6% were male, and 64% came from urban areas with predominantly low socioeconomic levels. Men showed a threefold increased risk of advanced OCSCC (95% CI: 1.3–6.8), while patients referred to pain clinics exhibited a 19.5 times higher risk (95% CI: 2.3–159.5). Patients in the subsidized health system or without health insurance had 2.6 (95% CI: 1.07–6.3) and 2.7 times (95% CI: 1.17–6.4) higher risks, respectively. **Conclusions:** This study found that male patients, referrals to pain clinics, and subsidized or no health system affiliation significantly increased the risk of advanced OCSCC.

## 1. Introduction

Oral cavity cancer, a subtype of head and neck cancer, remains a significant global health challenge due to its high incidence and mortality rates [1]. Globally, it is estimated that nearly 20 million new cancer cases occurred in 2022, with oral cavity squamous cell carcinoma (OCSCC) accounting for a notable proportion of these diagnoses. According to the latest data from the International Agency for Research on Cancer (IARC), the global cancer burden continues to increase, and projections suggest this trend will persist, with an expected 35 million new cases of cancer by 2050, highlighting the need for enhanced prevention and early detection efforts [2]. Over 90% of oral cavity cancers originate in squamous tissues, leading to their classification as OCSCC [1,3]. Incidence and mortality rates for OCSCC vary markedly by region, with Asia reporting the highest burden, comprising 64.2% of diagnosed cases and 73.3% of deaths related to the disease [1,4].

OCSCC is a cancerous growth originating from squamous epithelial cells that line the oral cavity, encompassing the lips, palate, tongue, upper and lower gums, and buccal mucosa, among other regions [5]. The primary risk factors for OCSCC include tobacco and alcohol consumption. Additionally, socioeconomic factors, such as inadequate dental hygiene and limited access to healthcare services, impact the timely detection and treatment outcomes [1,5]. Chronic discomforts, such as sharp teeth and poorly fitting dentures, as well as a poor diet, are all contributing risk factors [5]. Moreover, the clinical presentations of oral cancer, along with the side effects of treatment, can significantly affect the overall quality of life for patients. They may experience considerable challenges in speaking, swallowing, maintaining cosmetic appearance, sensory impairment, and enduring persistent pain [6]. Pain is a common symptom of OCSCC, particularly in the later stages of the disease. As the cancer progresses, it can invade nearby structures, which can also contribute to pain. Pain associated with OCSCC can vary in intensity and it is important to note that pain is not always present in the early stages of OCSCC, and some individuals may not experience pain until the disease has advanced [5,6]. Regarding this, a Brazilian study revealed that cancer patients predominantly experienced moderate to severe pain, which was correlated with disease stage and significantly impacted their quality of life and functional capacity [7].

Access to a healthcare system is a critical factor that influences the early diagnosis of OCSCC. Moreover, complex health system policies have a direct impact on health system management, potentially influencing the behavior and practices of both patients and healthcare workers [8]. Findings support the notion that enhanced public awareness of oral cancer and its symptoms, coupled with ongoing investments in public information initiatives, will encourage individuals exhibiting symptoms to seek consultation with a primary healthcare provider, thereby aiding in the early identification of the disease. The early detection of oral cancer should be prioritized over screening programs aimed at identifying the disease during its asymptomatic phases [9]. Low socioeconomic status has also been associated with both direct and indirect health outcomes in countries with public healthcare [10]. Individuals of lower socioeconomic status may face challenges such as difficulty taking time off work or affording childcare to receive care or recover from invasive procedures, and underserved neighborhoods may lack physicians and healthcare resources or adequate transportation options for residents to seek care or referrals [10,11,12]. Patients with common cancers from lower socioeconomic backgrounds were also significantly more prone to have their cancer diagnosed at later stages compared to those with higher socioeconomic status [10,13]. Therefore, systemic inequalities and a lack of resources may contribute to inferior outcomes for individuals with low socioeconomic status [10,14]. A similar trend has been observed in South America. The 110,162 deaths from oral cancer recorded in the official Brazilian health system between 1996 and 2019 revealed that oral cancer mortality rates differ by region in Brazil. Moreover, except for the Southeast, the National Oral Health Policy was not associated with a reduction in oral cancer mortality rates in Brazil. The pattern’s dynamics and the largest annual percentage change may be related to poorer socioeconomic conditions in the North, Northeast, and Midwest, suggesting difficulties in accessing oral healthcare services across the healthcare network [15].

It is widely recognized that healthcare fragmentation, a significant contributor to delays in cancer diagnosis and treatment, contributes to high mortality rates in Latin America, particularly among disadvantaged populations. Regrettably, Latin American healthcare systems are characterized by limited access to health services and inadequate coordination of care among multiple providers [16]. Research conducted in Latin America has shown that delays in diagnosis occur [17,18] and are associated with financial, geographic, and organizational barriers to access, such as low suspicion for cancer in primary care and limited coordination of care across different levels of the healthcare system [19,20]. The population groups most affected include those with low socioeconomic status residing in large urban slums, rural residents, indigenous peoples [20], and those in Colombia’s subsidized healthcare system (informal employment) [21], resulting in significant disparities in early diagnosis [22]. In this context, recent findings underscore the urgent need for additional research on oral cancer prevention, and it was confirmed that abandoning tobacco, alcohol, and areca nut consumption leads to a substantial reduction in oral cancer risk [23].

Considering all the background information previously presented, the objective of this study is to assess the influence of health system affiliation and pain manifestation in patients with advanced OCSCC in a Latin American population. We hypothesize that disparities in health system affiliation and early pain presentation impact both the stage of diagnosis and the outcomes of OCSCC in these patients. The null hypothesis (H0) states that there is no significant association between health system affiliation or pain presentation and the stage or outcomes of OCSCC in this population. In contrast, the alternative hypothesis (Ha) posits that health system affiliation and early pain presentation are significantly associated with later stages of diagnosis and poorer outcomes in advanced OCSCC. Specifically, this study aims to (1) evaluate the relationship between health system affiliation and diagnostic stage in OCSCC, (2) determine the prevalence of pain at the time of diagnosis and its correlation with disease stage, and (3) investigate the combined effect of health system affiliation and pain manifestation on the prognosis of OCSCC patients.

## 2. Materials and Methods

### 2.1. Population and Sample

Under an empirical–analytical approach, a retrospective cohort quantitative study was conducted through the secondary analysis of data extracted from patients’ medical records. Retrospective data were accessed for research purposes from 1 July 2015 to 30 May 2016, including data from the past 19 years. Thus, the population consisted of the medical records of patients with OCSCC treated in the maxillofacial surgery and stomatology unit of a public hospital in Medellín, Colombia. To ensure comprehensive analysis, all eligible medical records were included in the study, avoiding the need for a sampling design. This approach allowed us to leverage the full scope of available data, maximizing statistical power and minimizing potential selection bias associated with sampling.

### 2.2. Selection Criteria

All patient records were included for those who consulted, were diagnosed, and treated with OCSCC at the hospital San Vicente Fundación in Medellín, Colombia, during two decades, patients for whom information was referenced in the maxillofacial surgery and stomatology unit of the hospital. Medical records of patients with tumor lesions in the mouth with a diagnosis other than OCSCC and clinical records that did not allow the evaluation of the variables of interest were excluded.

### 2.3. Studied Characteristics

Sociodemographic variables were evaluated, including age, sex, educational level (Primary Education, Secondary Education, Technical/Higher Education), employment (formal, informal, unemployed), residential (rural/urban), marital status, smoking habit (yes/no), alcohol consumption (yes/no), family group (number of family members), socioeconomic level (low, medium, high) and health system affiliation (contributory, subsidized, none).

Residential status was classified according to the guidelines provided by the National Administrative Department of Statistics of Colombia (DANE; https://www.dane.gov.co, accessed on 15 May 2016). Urban areas typically refer to cities and towns with well-developed infrastructure, high population density, and availability of essential services. Rural areas are defined as regions outside these urban centers, often characterized by lower population density, limited access to healthcare and education, and a reliance on agriculture and other primary sector activities.

The family group variable refers to the number of individuals living in the same household. This categorization helps in understanding the household’s socioeconomic dynamics and potential impact on healthcare access and disease progression.

Socioeconomic levels in Colombia are determined by the System for Identifying Potential Beneficiaries of Social Programs (SISBEN) or similar social stratification tools. These classifications consider income, living conditions, education level, and access to basic services. A low socioeconomic level typically includes households with limited financial resources and access to services, medium level includes households with moderate resources, and high level includes households with substantial financial resources and access to high-quality services.

Contributory systems typically require individuals to make contributions to access healthcare services, while subsidized systems provide healthcare services at reduced or no cost to those who qualify based on income or other criteria. Those with no affiliation may have to pay out-of-pocket for healthcare services. Formal workers typically refer to individuals who are employed in jobs that are officially recognized and regulated by the government. These jobs often come with benefits such as health insurance, retirement plans, and paid leave. Formal workers also usually pay taxes on their income. Informal workers are those who work in jobs that are not officially recognized or regulated by the government. These jobs often do not come with benefits, and informal workers may not pay taxes on their income. Examples of informal workers include street vendors, domestic workers, and day laborers.

Clinical variables such as lesion site, time of onset of signs and symptoms, family history of cancer, time of treatment initiation, treatment delay time, TNM staging system for cancer (stages I, II, III, IV) [22], received treatment (surgery, radiotherapy, chemotherapy, or referral to pain clinic), recurrence, and retreatment were also considered. Recurrence was defined as the return of cancer after a period of remission. Specifically, it refers to the reappearance of cancer cells at the primary site (local recurrence), in the regional lymph nodes (regional recurrence), or in distant organs (distant recurrence) after the initial treatment has achieved a complete or partial response [24]. The outcome variable used was the advanced stage of the disease determined by stages III and IV of the TNM classification [22].

All the information was personally collected by one of the researchers and stored in a tool designed for this purpose. In this retrospective cohort study, the cohort’s experience over time was reconstructed using truthful, reliable, and verifiable information stored in the institution’s medical records archive, which allowed for the collection of the necessary data for analysis and obtaining results.

### 2.4. Statistical Analysis

Initially, a descriptive (univariate) analysis was performed using absolute frequencies, relative frequencies, and summary measures for sociodemographic and clinical variables. Subsequently, a bivariate analysis was conducted, taking the outcome of interest (advanced stage yes/no) as a reference to determine the association and magnitude of this outcome with the sociodemographic and clinical variables of interest using the chi-square test of independence for qualitative variables accompanied by the measure of association and the relative risk (RR), along with its 95% confidence interval for this indicator. In the quantitative variables, the normality of the outcome categories was determined using the Shapiro–Wilk test. Since the normality of the data was not confirmed, the Mann–Whitney U test was used to differentiate average ranks.

In constructing the logistic regression model, candidate variables were selected using the Hosmer–Lemeshow criterion (*p* < 0.25) based on biological plausibility. To meet the model assumptions, we evaluated the distribution of residuals, applying tests for normality and visually inspecting residual plots. A stepwise selection process was then used to construct the explanatory model. Data processing and analysis were performed using SPSS^®^ version 21.

This research was approved by the Ethics Committee of the Faculty of Public Health of the University of Antioquia, Medellín, Colombia (Record 121-15). Retrospective data were accessed for research purposes and the authors did not have access to information that could identify individual participants during or after data collection (e.g., names and identification numbers were anonymized).

## 3. Results

In this study, 233 patients with OCSCC were analyzed, of which 196 corresponded to an advanced stage of the disease. It was found that 53.6% of the patients were men, and the average age of all the patients was 63 ± 13 years. It was also found that 64% of the patients came from urban areas, and had a predominantly low socioeconomic level, with a predominance of primary studies when considering the level of education. Note that there were no significant differences in the marital status of the patients between married and unmarried, and as for the occupation, most were unemployed. Moreover, more than half of the patients were affiliated with the subsidized health system (Table 1).

After the initial consultation at the institution, the average time to confirm the diagnosis through histopathological study was 7.2 ± 7.7 days, and to initiate treatment was 45.2 ± 44 days. A large proportion of patients reported being smokers, while more than half of them reported alcohol consumption. The tongue was the most frequent anatomical site for the presentation of OCSCC, and 84.1% of all patients had an advanced stage of the disease. Combined treatment with surgery, radiotherapy, and chemotherapy was performed most frequently, with a recurrence rate of 9.4%, while 12.4% of patients underwent retreatment (Table 2).

Although normality was not confirmed by the Shapiro–Wilk test, quantitative data are presented as mean ± SD to facilitate comparison with existing literature. Non-parametric tests were used in the analysis to account for deviations from normality.

In Table 3, it can be observed that 91.2% of patients with advanced OCSCC were men, presenting a risk of 1.2 times compared to women suffering from the pathology (*p* < 0.001). It was also found that the risk of the advanced stage of the disease was 1.2 times higher if the patient did not have an affiliation or belonged to the subsidized regime with statistical significance (*p* < 0.02). The patient’s area of residence, level of education, marital status, occupation, and socioeconomic level were not statistically associated with the advanced stage of cancer, although the relative risk was higher in patients from rural areas, with primary education, and with a low socioeconomic level.

It was found that the highest proportion of patients with advanced carcinoma were those who received referral to the pain clinic as treatment (97.5%). These patients had 1.5 times the risk of developing the disease compared to those who received only surgery, with statistical significance (*p* < 0.0001). Similarly, those who received a combination of surgery, radiotherapy, and chemotherapy as treatment had 1.3 times the risk of those who received only surgery (*p* < 0.001). The smoking habit, alcohol consumption, family history of cancer, patients who received retreatment, and those who had recurrence were not statistically associated with the advanced stage of cancer, although the relative risk was higher in smokers, alcohol consumers, patients with a family history of cancer, those who received retreatment, and those with recurrence. Similarly, taking the anatomical site of the lesion as a reference, the risk of the advanced stage of the disease was higher if the patient had the disease in the tongue, which was statistically significant (*p* < 0.04) (Table 4).

To determine the variables that best explain the advanced stage of OCSCC, an explanatory logistic regression model was constructed (Table 5). The variables sex, affiliation to the health system, and treatment received explained 21.4% of the variability in the advanced stage of the disease (Nagelkerke R-squared 0.214). Significantly, men experienced 3 times more risk of presenting an advanced state of OCSCC. Moreover, the variable reference to the pain clinic had a higher risk of advanced cancer (19.5), while regarding the health system, those who belonged to the subsidized system or did not have a health system also had a higher risk of advanced cancer (2.6 and 2.7, respectively).

## 4. Discussion

In this study, we analyzed 233 patients with OCSCC at a national public hospital in Medellín, Colombia, a referral center for treating cancerous pathologies. Notably, this study provides the first epidemiological data on OCSCC in Medellín, adding novelty and significance by focusing on a previously unstudied patient cohort in this location. The study aimed to examine the disease progression at the time of consultation, hypothesizing that a substantial portion of patients would present at advanced stages due to delays in seeking medical attention. Our findings supported this hypothesis, as the average disease progression time was 11.2 months, with 196 patients (84.1%) presenting at advanced stages, corroborating existing literature that prolonged delays are a critical factor for advanced disease [25]. These findings are concerning because they greatly reduce the potential for curative treatments, emphasizing the need for timely intervention in similar patient populations.

Our study reveals that delays in diagnosing oral cancer are multifaceted, involving patient-related, provider-related, and systemic factors. We observed that patients often delay seeking care, averaging 104.9 days after initial tumor symptoms appear [26]. This is likely due to the asymptomatic nature of early-stage tumors and the inaccessible locations of some lesions, making self-detection challenging [25]. Furthermore, healthcare providers, including general practitioners and dentists, often lack awareness of early-stage carcinoma signs, contributing to diagnostic delays [25,27]. Public health services, which are strained by high patient volumes and limited resources, also play a significant role in these delays [15,25].

Epidemiological studies show that tobacco is the main risk factor for the development of oral cancer, as it contains over 300 carcinogenic components that are converted into reactive metabolites capable of interacting with DNA [27]. In this cohort, as well as in reports from the literature [28,29], over 80% of patients were observed to be smokers. While our study could not assess specific patient behaviors or diet due to limited data, we did evaluate smoking and alcohol consumption, which are well-established risk factors. Further research with comprehensive lifestyle data could provide more insights into additional behavioral contributions to OCSCC.

As observed in this cohort, the presence of OCSCC primarily on the tongue is confirmed by other studies [30,31]. Oral tongue squamous cell carcinoma is increasingly recognized as a distinct biological entity compared to cancer affecting other oral sites, exhibiting a more aggressive nature, and typically associated with a higher metastatic rate [32]. The tendency for subclinical occult nodal metastasis in early tongue cancer has been extensively studied [31].

As noted in the current investigation, oral cavity cancer typically impacts older males with a background of tobacco and alcohol use. Research has demonstrated that patient survival time decreases in an orderly and stepwise manner with increasing age groups [33]. A study conducted in Brazil revealed that age significantly influences mortality from oral and oropharyngeal cancer. The risk of mortality begins to rise at 40 years for men and 55 years for women, and an overall period effect was observed [34]. Additionally, research has indicated that younger patients experience higher survival rates in comparison to their older counterparts [33]. However, the impact of age on the prognosis of OCSCC remains a topic of debate [33,34]. While various studies have produced different findings, they have not been able to provide a detailed explanation of the etiology and pathological mechanism. Recent analyses suggest that younger patients are more likely to undergo surgery and triple therapy, indicating a tendency to opt for more aggressive treatments [33].

As observed in this cohort, patients referred to the pain clinic presented a more advanced stage of carcinoma (97.5% and a 1.5 times higher risk of developing OCSCC). In this context, it has been documented that pain is a common symptom among individuals with oral cancer, constituting 30–40% of their initial complaints [35]. Despite pain being the primary concern, it typically emerges only after the lesions have significantly progressed, prompting the patient to seek medical attention. Early-stage carcinomas often go unnoticed due to their asymptomatic nature [36]. In later stages and larger lesions, symptoms can vary from mild discomfort to severe pain, especially on the tongue. Other symptoms may include ear pain, bleeding, tooth displacement, breathing difficulties, speech issues, swallowing difficulties, problems with dental prostheses, trismus, and paresthesia [37]. It has been extensively shown that nerves play a significant role in tumor-associated microenvironments. Given the typical innervation of the oral cavity and the distinct presence of cancer-associated pain in OCSCC, sensory nerves may have a prominent role in the OCSCC–nerve microenvironment. As the most prevalent neuropeptide in the trigeminal ganglion, calcitonin gene-related peptides have a dual effect on cancer progression and cancer-associated pain across different cancer types [38]. In our study, referral to the pain clinic serves as an indicator of advanced disease rather than a preoperative measure. Patients referred to the pain clinic typically presented with significant pain symptoms, which are often associated with more advanced stages of OCSCC. This pattern suggests that severe pain reflects the disease progression at the time of diagnosis, rather than a contributing factor to delayed diagnosis. Thus, the high risk of advanced OCSCC observed in patients referred to pain clinics highlights the critical role of early detection and intervention in managing OCSCC, as severe pain can indicate a more progressed disease state.

A highly pertinent finding in this study was the association between health system affiliation and the risk of presenting a more advanced stage of carcinoma. Specifically, individuals without health system affiliation or those belonging to a subsidized regime were found to have a significantly higher risk of presenting an advanced stage of carcinoma. A combination of social determinants, such as advanced age, male gender, low socioeconomic status, and absence of private insurance, has likewise been linked to a poorer prognosis among individuals diagnosed with cancer [15,39,40]. Patients with insurance plans other than private ones were also found to have poorer short-term postoperative outcomes. Specifically, those who were older, covered by Medicaid, and residing in urban or rural areas were less likely to be readmitted within 30 days of surgery [41]. Conversely, increased readmissions were linked to treatment in non-academic settings. These same factors were also associated with advanced disease at the time of diagnosis and overall prognosis [15,41]. Recent research has indicated that patients often face worse outcomes due to obstacles in accessing treatments promptly [15,42]. An analysis of a national cancer database revealed that the inferior short-term outcomes observed are likely due to limited access to treatment, especially among individuals with low socioeconomic status, non-private insurance, and physical barriers within the healthcare facility [41]. A strong caregiver and support network can significantly enhance a patient’s resilience, which has been linked to improved quality of life and surgical outcomes [43]. More structured forms of social support, such as cancer support groups and online forums, have demonstrated similar effectiveness. However, patients and their caregivers often do not seek out these resources until the disease has progressed significantly [44]. Early engagement with social support may help to mitigate adverse treatment outcomes in patients with cancer [41].

An intriguing Brazilian national study utilized the three-delay model to propose that a combination of factors would be necessary to decrease mortality rates for oral cancer. This model offers a valuable framework for examining the factors that affect the timeliness of care. The first delay explores how socioeconomic and cultural factors impact a person’s decision to seek help. The second delay is largely influenced by structural considerations needed to access the healthcare institution. The third delay encompasses factors within the healthcare facility, such as service availability and quality [15]. Another study of a cancer database from a high-income country underscores the importance of integrating social services into routine clinical practice. After a cancer diagnosis, individuals would benefit from being informed by patient navigators about the support groups available in their healthcare system. These efforts are especially critical in regions with significant uninsured populations, high poverty rates, and a higher comorbidity index [41].

Although public resources allocated to health systems in low- and middle-income countries, such as those in Latin America, are limited, an effective health system for the prompt diagnosis and treatment of oral cancer should ideally possess several key characteristics. These include a robust primary care infrastructure, which is essential for the early detection and referral of patients with oral cancer. This infrastructure should be supported by trained healthcare providers at the community level who can identify suspicious lesions and refer patients for further evaluation. Access to basic diagnostic tools, such as oral examination kits, biopsy equipment, and basic imaging technologies, is also crucial for diagnosing oral cancer in these settings. Furthermore, healthcare providers at all levels, including primary care workers, nurses, and physicians, should receive training in oral cancer detection and management. Basic treatment facilities, such as surgical centers and radiation therapy units, should be available at the district or regional level to provide timely treatment to patients with oral cancer. To ensure continuity of care and monitoring of outcomes, oral cancer services should be integrated into existing health systems, such as primary care networks and cancer registries. Additionally, services for oral cancer diagnosis and treatment should be affordable and accessible to all segments of the population, including those in rural and remote areas.

The relationship between healthcare system affiliation and pain clinic management is critical in understanding patient outcomes in OCSCC. Patients who are uninsured or affiliated with subsidized healthcare systems may face barriers to accessing comprehensive care, including timely referrals to pain management services. Our findings indicate that these patients are at a higher risk of presenting with advanced OCSCC, suggesting that socioeconomic factors can influence the likelihood of receiving adequate pain management. This interplay may create a cycle where inadequate access to pain management exacerbates patient suffering and disease progression, underscoring the need for integrated healthcare policies that ensure equitable access to pain management services for all patients, regardless of their insurance status.

This study’s major strength lies in its focus on a previously unstudied population in Medellín, providing valuable insights into OCSCC in a Latin American context. Additionally, our findings regarding healthcare affiliation and advanced disease stages underscore the critical role of healthcare access in cancer prognosis, particularly in low- and middle-income countries.

Although cohort studies are observational studies with the highest level of evidence, the retrospective analysis of the cohort reviewed here may not be exempt from some biases related to the control of certain variables; however, the multivariate regression analysis conducted allowed us to somehow control this type of bias. Nevertheless, more studies are required to evaluate especially the influence that health systems have on the presence of advanced stages of OCSCC, particularly in low- and middle-income countries. We acknowledge that the sample size for the Stage I/II comparator group is relatively small compared to the advanced-stage group. This discrepancy reflects the real-world distribution of OCSCC stages within our study population. Despite this limitation, we employed rigorous multivariate regression analysis and found significant and consistent results. Another limitation of our study is the lack of ethnicity data, which precluded analysis of potential ethnic disparities in OCSCC progression and healthcare access. The lack of data on some behavioral factors (e.g., diet and physical activity) also limit the generalizability of our findings. The study did not capture information beyond smoking and alcohol consumption, which could have further contextualized risk factors.

The association between health system affiliation and advanced carcinoma stages is particularly noteworthy, suggesting that patients without private insurance or those in subsidized health systems face significant barriers to timely care. This highlights the importance of equitable access to healthcare resources, including early detection and intervention programs for oral cancer, especially for underserved populations. Future research should explore strategies to address these disparities, perhaps by implementing primary care infrastructure enhancements and incorporating cancer support services within existing health systems.

Further studies are essential to evaluate how health systems influence OCSCC presentation stages, particularly in low- and middle-income countries, where access to healthcare may be limited. Moreover, integrating social support networks early in the treatment process could mitigate adverse outcomes, as suggested by recent literature [41,43,44]. Efforts to incorporate the three-delay model, which considers socioeconomic, structural, and institutional factors, could serve as a valuable framework for reducing diagnostic delays and improving patient outcomes [15].

By addressing these findings, we hope this study will assist healthcare professionals and policymakers in designing targeted interventions that can reduce diagnostic delays and improve survival rates for patients with OCSCC, especially in settings with limited healthcare resources.

## 5. Conclusions

This study showed that men and individuals referred to pain clinics, as well as those without health system affiliation or with a subsidized affiliation, are at an increased risk of experiencing an advanced stage of OCSCC. It is essential to establish effective health systems for the early diagnosis and treatment of oral cancer in low- and middle-income countries.

## Figures and Tables

**Table 1:** Sociodemographic characteristics of the studied population.

Characteristic		N	%	95% Confidence Interval	*p*-Value
Sex	Female	108	46.4	39.8–52.9	0.26
	Male	125	53.6	47.0–60.2	
Residential	Urban	149	63.9	57.3–70.3	<0.001
	Rural	84	36.1	29.7–42.7	
Education Level	Primary	119	51.1	44.6–57.6	<0.001
	Secondary	40	17.2	12.4–22.9	
	Technical/Higher	5	2.1	0.7–4.5	
	None	17	7.3	4.4–11.2	
	Not reported	52	22.3	17.4–27.9	
Marital status	Married	111	47.6	41.1–54.3	0.47
	Single	122	52.4	45.7–58.9	
Employment	Formal	6	2.6	0.9–5.5	
	Informal	111	47.6	41.1–54.3	
	Unemployed	116	49.8	43.2–56.4	<0.001
Health system affiliation	Contributory	50	21.5	16.4–27.3	
	Subsidized	130	55.8	49.2–62.3	<0.001
	None	53	22.7	17.5–28.7	
Socioeconomic level	Low	169	72.5	66.8–77.8	<0.001
	Medium	64	27.5	22.2–33.2	
	High	0	0	0	

**Table 2:** Clinical characteristics of the studied population.

Characteristic		N	%	95% Confidence Interval	*p*-Value
Smoking	Smoker	194	83.3	77.8–87.8	<0.001
	Non-smoker	39	16.7	12.2–22.2	
Alcohol consumption	Yes	129	55	48.3–61.5	0.10
	No	104	45	38.5–51.7	
Anatomic location of the lesion	Tongue	72	30.9	25.0–37.3	0.10
	Palate	39	16.7	12.2–22.2	
	Mouth floor	42	18.0	13.3–23.6	
	Maxillary and mandibular bone	33	14.2	10.0–19.3	
	Alveolar ridge and retromolar trigone	28	12.0	8.1–16.9	
	Lip and cheek	19	8.2	5.0–12.4	
Family history of cancer	Yes	17	7.3	4.3–11.4	
	No	216	92.7	88.5–95.7	<0.001
Stage of the disease	Early	37	15.9	11.4–21.2	
	Advanced	196	84.1	78.8–88.6	<0.001
Treatment	Surgery	46	19.7	14.8–25.4	0.47
	Radiation therapy and chemotherapy (palliative treatment)	54	23.2	18.0–29.1	
	Surgery, radiation therapy and chemotherapy	92	39.5	33.2–46.1	
	Clinical pain remission	41	17.6	13.0–23.1	
Retreatment	Yes	29	12.4	8.5–17.4	
	No	204	87.6	82.6–91.5	<0.001
Recurrence	Yes	22	9.4	6.0–14.0	
	No	211	90.6	86.0–94.0	<0.001

**Table 3:** Distribution of demographic variables according to the stage of oral squamous cell carcinoma.

Variable		Advanced Stage	Early Stage	% Advanced Stage	*p*-Value	RR *	95% Confidence Interval
Sex	Male	114	11	91.2	0.001	1.2	1.1–1.4
	Female	82	26	75.9	1		
Residential	Rural	73	10	88.0	0.226	1.1	1.0–1.2
	Urban	122	27	81.9	1		
Education Level	Primary	114	22	83.9	0.555	1.1	0.9–1.2
	Secondary	36	9	80.0	1		
Marital status	Single	103	19	84.4	0.9	1.0	0.9–1.1
	Married	93	18	83.8	1		
Employment	Unemployed	95	21	81.9	1.000	1.0	0.7–1.4
	Informal	96	15	86.5	1.000	1.0	0.7–1.5
	Formal	5	1	83.3	1		
Health system affiliation	None	45	8	84.9	0.02	1.2	1.0–1.4
	Subsidized	114	16	87.7	0.02	1.2	1.0–1.4
	Contributory	37	13	74.0	1		
Socioeconomic level	Low	142	25	85.0	0.5	1.1	0.9–1.2
	Medium	52	12	81.2	1		

* Relative risk.

**Table 4:** Distribution of clinical variables according to the stage of oral squamous cell carcinoma.

Clinical Variable		Advanced Stage	Early Stage	% Advanced Stage	*p*-Value	RR *	95% Confidence Interval
Smoking	Smoker	167	27	86.1	0.068	1.1	0.9–1.4
	Non-Smoker	29	10	74.3	1		
Alcohol consumption	Yes	111	16	87.4	0.117	1.1	1.0–1.2
	No	83	21	80.0	1		
Anatomic location of the lesion	Tongue	55	17	76.3	0.04	0.9	0.7–1.0
	Palate	33	6	84.6	0.563	0.9	0.8–1.1
	Mouth floor	37	5	88.1	1.000	1.0	0.9–1.1
	Maxillary/other sites	71	9	88.8	1		
Family history of cancer	Yes	16	1	94.1	0.487	1.1	0.1–1.3
	No	180	36	83.3	1		
Treatment	Clinical pain remission	40	1	97.5	0.0001	1.5	1.2–1.8
	Surgery, radiation therapy and chemotherapy	126	20	86.3	0.001	1.3	1.1–1.6
	Surgery	30	16	62.5	1		
Retreatment	Yes	26	3	89.6	0.587	1.1	0.9–1.2
	No	170	34	83.3	1		
Recurrence	Yes	21	1	95.4	0.216	1.2	1.0–1.3
	No	175	36	82.9			1

* Relative risk.

**Table 5:** Multivariate logistic regression model for advanced-stage oral squamous cell carcinoma.

Variable	Beta	Total Error	Wald	Degrees of Freedom	*p*-Value	Exponential B	95% Confidence Interval
Sex	1.131	0.407	7.730	1	0.005	3.099	1.396–6.879
							
Surgery			11.845	2	0.003		
Treatment combination	1.106	0.414	7.147	1	0.008	3.022	1.343–6.798
Clinical pain remission	2.969	1.073	7.657	1	0.006	19.5	2.378–159.561
							
Contributory			4.577	2	0.101		
None	0.918	0.544	4.590	1	0.002	2.749	1.173–6.406
Subsidized	0.956	0.453	4.453	1	0.035	2.602	1.070–6.326
Constant	−0.379	0.465	0.663	1	0.415	0.685	

## Data Availability

The datasets used and analysed during the current study are available from the corresponding author upon reasonable request.
